# A Concise Profile of Gallic Acid—From Its Natural Sources through Biological Properties and Chemical Methods of Determination

**DOI:** 10.3390/molecules28031186

**Published:** 2023-01-25

**Authors:** Dorota Wianowska, Małgorzata Olszowy-Tomczyk

**Affiliations:** Department of Chromatography, Institute of Chemical Sciences, Faculty of Chemistry, Maria Curie-Skłodowska University in Lublin, Pl. Maria Curie-Skłodowska 3, 20-031 Lublin, Poland

**Keywords:** gallic acid biological properties, LC and GC gallic acid determination, gallic acid isolation methods

## Abstract

Nature is a valuable source of anti-oxidants that have a health-promoting effect by inhibiting various undesirable changes leading to cell degradation and, consequently, potential disease ailments. One of them is gallic acid which has been used as a healing agent since ancient times. Currently, due to various beneficial properties, this compound is considered to be one of the main phenolic acids of great importance in numerous industries. It is commonly used as a substance protecting against the harmful effects of UV radiation, an astringent in cosmetic preparations, and a preservative in food products. Therefore, gallic acid is now deemed essential for both human health and industry. Increasingly better methods of its isolation and analysis are being developed, and new solutions are being sought to increase its production. This review, presenting a concise characterization of gallic acid, updates the knowledge about its various biological activities and methods used for its isolation and determination, including chromatographic and non-chromatographic methods.

## 1. Introduction

**Figure 1:**
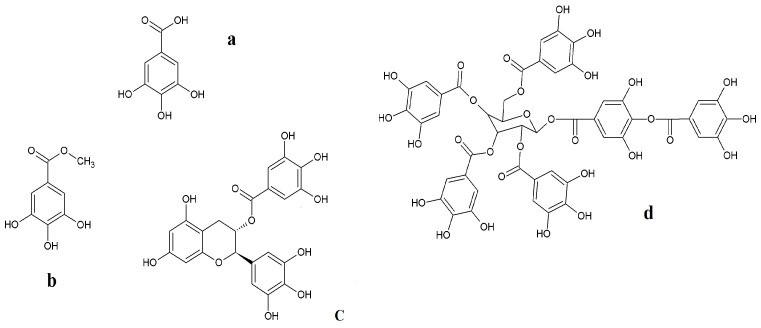
Chemical structure of gallic acid (**a**) and its derivatives: gallic acid methyl ester (**b**), (-)-epigallocatechin gallate (**c**), and hydrolysable tannin (**d**).

Gallic acid (3,4,5-trihydroxybenzoic acid), discovered by Carl Wilhelm Scheele in 1786 while studying a grey precipitate with sour taste formed in oak apple extract, was initially underestimated, and now is considered one of the main phenolic acids of great importance in numerous industries [1,2]. It is found in many plants of the families *Anacardiaceae*, *Fabaceae*, and *Myrtaceae*, as well as in fungi of the genus *Termitomyces* [3], in the form of both free and more complex molecules (see Figure 1). Of the latter, the most commonly known is the group of hydrolysable tannins, the so-called gallotannins, capable of precipitating proteins and forming complexes with toxic metal ions, reducing their bioavailability in the environment. Indeed, this is not the only positive role of this compound.

Currently, in addition to its strong anti-oxidant activity, gallic acid (GA) is also attributed with anti-cancer, anti-HIV, anti-ulcer, anti-inflammatory, anti-microbial, and anti-fungal properties [2,4,5,6,7,8,9,10,11]. Recently, reports on the possible involvement of the acid in reducing neuronal damage and brain amyloid neuropathology, characteristic of Alzheimer’s disease, and improving the cognitive function by scavenging free radicals and inhibiting Aβ oligomerization, have greatly intensified research on this compound and general interest in its properties [12]. It is worth emphasizing that interest in the GA properties goes beyond the medicinal aspects [13]. Resulting from the studies by Fernandes and Salgado [1], the first commercial application of GA was associated with its chelating ability. This property is exploited in the skin and leather industry. Gallic acid is applied as an ingredient of developer in photography and printing inks [14]. It also serves as a precursor for the commercial production of the anti-microbial drug, trimethoprim. Additionally, owing to its ability to neutralize free radicals, it is used as a preservative to prevent oxidation of food and beverages [15]. This compound is used as a substance for protecting against the harmful effects of UV radiation as well as an astringent in cosmetic preparations. In the brewing and wine industries, GA is applied as a clarifying agent. A very important application of gallic acid is the production of food packaging. The inclusion of GA in fish gelatin film, which is protein-based, makes food packaging environmentally friendly and an alternative to help reduce the use of synthetic plastic materials. Moreover, it helps to increase the mechanical properties of the packaging (strength and stretchability of the film) as well as the anti-oxidant capacity. This new food packaging can be used as a food film wrapping for halal and kosher food, as it is made of fish gelatin. In addition, owing to GA, this packaging is characterized by anti-oxidant properties, which allows it to be used in another group of products [16]. With so many positive properties, it is not surprising that GA is considered essential for both human health and industry. Therefore, increasingly better methods of its isolation and analysis are being developed and new solutions are being sought to increase its production.

Despite a noticeable revival of interest in the properties of gallic acid, there are not many review papers on this compound in the literature. To the best of our knowledge, there is only one paper by Fernandes and Salgado, published in 2016 [1], on the chromatographic methods of its analysis, mainly by HPLC, but there is no broader consideration of other possible analysis techniques. In addition, an up-to-date summary of the biological properties of this compound is missing. This paper fills this gap by summarizing the current state of knowledge about the properties of GA and its relevance to the modern world, including different methods currently used for its isolation and analysis. As there is still room for improvement in the effectiveness of these methods, this review shows the directions that need to be taken to make these methods faster and more environmentally friendly.

## 2. Review Methodology

The present literature review was compiled by systematically collecting, reviewing and assembling information (1993 to 2022) from available online databases such as Google Scholar, Scopus, Web of Science, PubMed, and Science Direct. This comprehensive search was conducted using keywords (“biological properties of gallic acid”, “sources of gallic acid”, “gallic acid determination”, “gallic acid analysis”, and “gallic acid isolation”). The search was limited to the English language. In addition, the abstracts were pre-screened before studying the whole documents. The literature review was analyzed in-depth to summarize the general knowledge about gallic acid. The search results were checked individually by two authors.

## 3. Gallic Acid Properties, Occurrence, and Production

Gallic acid, identified by Carl Wilhelm Scheele, is a grey powder with a sour taste that effervesces in calcium carbonate solution, is well soluble in ethanol and turns litmus red. Nowadays, it is commonly known that a pure GA is a colorless, crystalline powder. Besides the fact that it is soluble in water, it can be also dissolved in alcohol, ether, and glycerol. It is practically insoluble in benzene, chloroform, and ether petroleum [17,18,19,20]. Its main chemical and physical characteristics are listed in Table 1.

GA occurs in nature mainly in the form of hydrolysable tannins. However, their amounts as dietary components are limited [21]. The main sources of this compound in the human diet are non-sugar galloyl esters of GA such as epigallocatechin gallate, which releases GA during heating. These compounds are quite commonly present in grapes, wines, mangoes, green and black teas, and even edible mushrooms. Table 2 shows the content of GA in various plant foods. It is worth noting that GA is not available in the form of typical supplements. However, in the marketplace, one can find many products which are additionally enriched with GA.

Gallic acid is formed in plants in the shikimate pathway, which provides aromatic amino acids that are precursors of numerous secondary metabolites such as: coumarins, alkaloids, lignans, or polyphenols, including GA. The exact mechanism of GA synthesis in higher plants is not known. For this reason, three alternative routes for its production have been proposed in the literature: (1) α-oxidation of 3,4,5-trihydroxycinnamic acid to GA, (2) hydroxylation of protocatechuic acid, and (3) direct dehydration of 3-dehydroshikimic acid to GA [1,31].

According to the data published in 2015 [32], the global demand for GA amounted to 8000 tons and this could not be satisfied from natural sources alone. A search for ways to source it started, which resulted in the commercial production of gallic acid from various inedible plants, such as: tara (*Caesalpinia spinosa* (Molina) *Kuntze*) fruit pods, *Terminalia chebula* seeds, sumac (*Rhus coriaria* L.) leaves, etc., as a result of the decomposition of tannic acid. In the release of gallic acid from the above-mentioned materials, the main role is played by the glycoprotein esterase, i.e., tannase. It is a very important microbial enzyme, especially from the point of view of commercial applications, as it is involved in the hydrolysis of esters and linkages in hydrolysable tannins such as tannic acid. This enzyme is produced by the fungi of the genera *Aspergillus, Penicillium, Fusarium* and *Trichoderma,* and the bacteria of the families *Bacillaceae, Corynebacteriaceae, Lactobacillaceae, Yersiniaceae, Enterococcaceae, Streptococcaceae,* and *Pseudomonadaceae*. According to the literature, currently the efficiency of gallic acid production using various inedible parts of plants and microorganisms exceeds 90% [15,17].

## 4. Biological Activity

Gallic acid is characterized by numerous biological properties. Nevertheless, the most characteristic is the anti-oxidant effect, which was decisive for undertaking the concise characterization of its pro-health effects. The following sections focus on the less obvious properties of GA, including anti-inflammatory, anti-microbial, anti-cancer, and others.

### 4.1. Anti-Oxidant Activity

The anti-oxidant activity of GA was determined by measuring its different abilities: the ability to neutralize an artificial radical (ABTS cation radical and/or DPPH radical), reduce metal ions (in the FRAP method), protect fluorescein (neutralization of peroxyl radicals), and inhibit the oxidation process (in a conjugated autoxidizable triene assay, the determination of lipid hydroperoxide value, and in a thiobarbituric acid reactance assay) [33,34]. Its strong anti-oxidant effect was confirmed in all of them.

As commonly known, the anti-oxidant capability of GA is related to the number of hydroxyl groups in the ring structure [35,36]. Moreover, the anti-oxidant action of GA should be associated with its ability to increase the activity of such enzymes as: dismutase superoxide, catalase, glutathione reductase, and glutathione peroxidase or with the elevation of non-enzymatic anti-oxidants (glutathione, vitamin C, and vitamin E) [37,38]. As reported in [39], GA is able to reverse Pb-induced oxidative damage. This is associated not only with its ability to scavenge ROS, such as superoxide anions, hydrogen peroxide, hydroxyl radicals, and hypochlorous acid, but also with its capacity to improve body anti-oxidant status (restoration of the activities of dismutase superoxide and catalase, whose function is deteriorated by Pb ions) [40,41]. In this way, the advantageous application of this anti-oxidant in Pb intoxications was proved. Additionally, its anti-oxidant activities are exhibited in the protection against DNA damage and lung injury due to oxidative stress. Moreover, GA possesses the ability of diabetic oxidative stress attenuation. 

In many papers on the assessment of the anti-oxidant properties of single substances or their mixtures, GA is used as the so-called standard anti-oxidant, i.e., a compound based on which the anti-oxidant properties of other compounds are determined [42,43]. Many phenolic compounds can be used as the standard. The criteria of its selection are related to stability, price, and/or solubility in the reaction environment characteristic of each method. The most important feature, however, is the composition of the tested sample and the standard similarity to the tested compounds. Thus, GA is used as the standard anti-oxidant in the DPPH method [44] or in the process of determining the sum of phenolic compounds in the so-called Folin–Ciocalteu method [45,46]. In these methods, the anti-oxidant activity of the test sample is expressed as gallic acid equivalent (GAE) in such units as μmol/g of sample [47] or mg of gallic acid equivalents (GAE)/100 g of sample (dry weight) [48]. In all cases, the results are calculated according to the gallic acid standard curve [49,50,51]. When choosing gallic acid as the standard anti-oxidant, it is not without significance that this compound provides the best response in many methods. For example, Antolovich et al. in [52], comparing the anti-oxidant activity of GA, uric acid, trolox, and ascorbic acid, four commonly applied anti-oxidants, using the TEAC (Trolox Equivalent Anti-oxidant Capacity), TRAP (Total Radical-Trapping Anti-oxidant Parameter), and the LDL (Low Density Lipoprotein) oxidation tests proved that gallic acid was the strongest anti-oxidant in all three systems and the relative activity of the remaining compounds depended on the system.

It should be emphasized that, despite the abundance of data demonstrating the anti-oxidant activity of GA, some of them are still controversial, proving the pro-oxidant effect of the compound. Based on the latest results, the issue of whether the compound is a potent anti-oxidant or a pro-oxidant remains debatable. This is a consequence of the fact that the phenolic properties switch from the anti- to pro-oxidant activity depending on their concentration, the presence of free transition metal ions, or their redox status [53]. In [54], the pro-oxidant activity of gallic acid was determined in a measuring system containing iron ions, proving that GA interacting with iron ions exhibits a pro-oxidant activity towards DNA and carbohydrates. This noxious behavior of GA is strongly dependent on the doses. At a low concentration of GA, the anti-oxidant activity was observed. When this compound is used in large doses, it causes induction of the apoptosis process [55]. Mard et al. [56] noticed that, at the concentration of 60 mg/kg (this was the concentration administered to the examined rats), GA was less effective than that of 30 mg/kg. The latter concentration was assumed to be an optimal concentration at which the compound exhibits the largest gastroprotective effect. This pro-oxidant activity of GA can be associated with the fact that small phenolic compounds are readily oxidized under some conditions. This fact can also be associated with the stability and reactivity of the phenoxyl radicals generated during the oxidation process of GA. Under conditions which cause the radical to undergo a reaction leading to a stable and non-harmful final product, the compounds exhibit anti-oxidant activity. When the formed radical takes part in other oxidation reactions, the compound exhibits pro-oxidant properties [57]. As mentioned above, the pro-oxidant activity of gallic acid depends on the concentration and presence of metal ions. Furthermore, Ph and structural features are responsible for these properties of gallic acid. At the alkaline Ph, GA is instable, which results in its auto-oxidation. This fact is associated with the production of reactive oxygen species and depletion of another anti-oxidant (for example, glutathione) in its presence [58].

### 4.2. Anti-Inflammatory Activities

Gallic acid is associated with a number of processes responsible for the reduction in pro-inflammatory factors in the human body [37]. One of them is reducing the expression and activity of enzymes (including inducible nitric oxide synthase and myeloperoxidase) responsible for inflammation. Another is the regulation of pro-angiogenesis factors, promotion of the angiogenesis process, or the inhibition of apoptosis parameters. It was also found that the GA dose-dependently reduces the disease activity index as well as macroscopic and microscopic damages (e.g., changes in the mucous membrane of the colon and stomach) [55,59]. In [60,61], it was shown that GA inhibits the release of lipopolysaccharide, stimulated nitric oxide, prostaglandin E2, interleukin-6 and cycloxxgenase-2 from macrophages during inflammatory processes. In [62,63], it was also revealed that GA plays an anti-inflammatory role by inhibiting the NF-Κb pathway considered to be a prototype pro-inflammatory signaling pathway in the body.

### 4.3. Anti-Dengue Properties

According to Suganthi and Ravi [64], dengue is a mosquito-borne viral infection that infects 50 to 100 million infants, children, and adults worldwide each year. The disease has a variety of clinical symptoms, from a fever known as the dengue fever to the so-called dengue shock syndrome. As follows from the data presented in [65] by Rothan et al., GA as the main compound of the *Vitris cinerea* extract is responsible for the great anti-dengue properties of the extract. The anti-dengue effect of GA is related to its ability to inhibit the production of infectious viral particles and to prevent virus entry into cells [65,66,67].

### 4.4. Anti-Platelet Activities

Platelet aggregation and activation are known to be the main reasons for atherosclerotic diseases [68]. Meanwhile, GA is considered to be an inhibitor of platelet and leukocyte aggregation, as well as P-selectin expression. Its activity is concentration-dependent. In addition, this compound is responsible for preventing the increase in intracellular calcium levels and reducing the phosphorylation process associated with this phenomenon.

### 4.5. Anti-Apoptotic Activities

Apoptosis is one of the natural biological processes of programmed and controlled destruction of own cells in the multi-cellular organism. This mechanism is needed and affects the proper development, homeostasis, and prevention of excessive, harmful multiplication of body cells. It can be induced by various stimuli and common signalling mediators [69], such as 6-hydroxydopamine or reactive oxygen species (ROS). As reported in [70], GA exhibits dose-dependent anti-apoptotic properties as it prevents the 6-hydroxydopamine-induced apoptosis (through its auto-oxidation) of dopaminergic cells. The cited paper also showed that the compound affects intracellular glutathione levels, ROS production, and Ca^2+^ influx, which independently indicates a protective effect of GA against apoptosis.

### 4.6. Anti-Microbial Activities

Owing to three hydroxyl groups in its structure, GA is regarded to be a toxic substance for micro-organisms. For example, in [8,71], it was proved that GA exhibits anti-microbial activity against: *Salmonella typhimurium, Escherichia coli*, *Staphylococcus aureus*, *Listeria innocua*, *Helicobacter pylori*, *Campylobacter spp*., and *Pseudomonas*. It is believed that GA possesses the ability of deterioration of bacterial cell membranes, which is responsible for irreversible changes in the permeability profile, rupture, pore formation, and decrease in negative surface charge. A consequence of this action is the leakage of essential intracellular constituents. According to [72], the anti-microbial activities of GA can also be associated with the effects of Ph as well as chelation of divalent cations. It is worth emphasizing that the anti-microbial properties of GA have been noticed, so that now this compound is used in the synthesis of silver nanoparticles coated with gallic acid. This combination is able to attach to microbes, disturbing the permeability and respiration functions, as well as penetrate through the microbial walls, causing serious damage; it can also interact with components containing sulfur and phosphorus, such as protein and DNA [73].

### 4.7. Anti-Tumor and Anti-Cancer Properties

Another particularly active area of research on the biological activity of GA concerns its ability to induce cell apoptosis in various types of cancers such as: lung, cervical cancer, oral squamous carcinoma, prostate cancer, melanoma, leukemia, lymphoma, colon cancer, pancreatic cancer, and breast cancer cells. Thus, by inhibiting tumor growth, GA exhibits anti-tumor activity. Its properties are associated with the effects of interfering with the generation of reactive oxygen species, disrupting the mitochondria function, regulation of apoptotic and anti-apoptotic proteins, suppression and promotion of oncogenes, and inhibition of matrix metalloproteinases [74,75]. 

In recent years, more and more attention has also been paid to the ability of GA to enhance the anti-cancer effect of drugs such as cisplatin, used in the treatment of cancer [75,76,77,78]. In this sense, the compound exhibits anti-cancer activity. The recently published research results of Khorsandi et al. [38] suggested that using infrared laser irradiation improves the anti-cancer properties of GA. According to the authors, human breast and melanoma cancer cells can be sufficiently destroyed in the presence of GA and low-level laser irradiation, and the mode of action is related to the induction of apoptosis and ferroptosis pathways. The latter pathway represents a new type of iron-dependent cell death that has been discovered in recent years.

Finally, it should be mentioned that, besides the above-mentioned activities, GA can act as an anti-depressant, anti-diabetic, anti-malarial, diuretic, cardioprotective, anti-viral, anti-fungal, anthelmintic anxiolytic, and anti-septic remedy [79,80,81,82]. It can promote wound healing. According to new reports, GA can also be used as an efficient agent in neurodegenerative diseases such as Alzheimer’s and Parkinson’s disease. As reported in [83], the great neuroprotective effects of gallic acid, both in vitro and in vivo, result from the regulation of anti-oxidant enzyme activities, neuroinflammatory cytokines, cytosolic Ca^2+^ concentration, and ROS generation. 

Gallic acid could prevent neuronal death and increase the learning and passive avoidance memory. This substance is also able to prevent the kidneys from nephrotoxicity induced by methotrexate (this is an anti-neoplastic agent that can be applied in the treatment of cancer and inflammatory diseases). During its application, GA causes a decrease in serum amyloid A (protein that is responsible for the deposition of amyloid in the tissues) [84]. Kim in [85] also demonstrated that GA exhibits anti-melanogenic properties due to its ability to inhibit tyrosinase and reduce melanin synthesis.

An objective view of the biological properties of GA requires at least pointing out that this compound can cause side effects. According to [86], GA can be responsible for a contractile and inhibitory decrease in vascular pressure in the aorta. It is possible that this compound also disturbs the action of drugs applied in the treatment of heart diseases. In the cited paper, the side effects of gallic acid were observed in the experiments on mice fed with food containing 0, 0.2, 0.6, 1.7, and 5% gallic acid for 13 weeks. The authors found that the administration of 0.6% or more gallic acid in males and 5% in females resulted in a decrease in hemoglobin concentration, hematocrit and red blood cell counts, and an increase in reticulocytes. In addition, the histopathological examination revealed hemolytic anemia. At the level of 1.7%, centrilobular liver cell hypertrophy, shown as an increase in the liver weight, was also observed.

## 5. Isolation and Determination of Gallic Acid 

The applicability of GA means that currently there are many papers dedicated to the isolation and analysis of this compound in various types of matrices. At the analysis stage, the currently preferred method of choice for the direct determination of GA in various types of samples is chromatography. In addition to chromatography, electrochemical methods and capillary electrophoresis are also quite commonly applied. All these methods require an appropriate sample preparation step, which allows not only the isolation of the analyte from a complex and complicated sample matrix, which enables direct determination, but can also significantly improve the analytical performance in terms of sensitivity, selectivity, and accuracy [87]. A summary of the literature review regarding the methods used to isolate and analyze GA in different types of samples is provided in Table 3.

### 5.1. Isolation of Gallic Acid

#### 5.1.1. Conventional Isolation Methods

The sample preparation procedure for GA determination is very often a multi-step process, the choice of which depends on the sample matrix type and final assay technique. Depending on the sample matrix type, different pre-treatment procedures are also required. Plants and agricultural wastes are usually dried and then ground or homogenized and passed through sieves to obtain a uniformly fine powder. The isolation of GA is also possible from fresh plant samples. However, a large moisture content can interfere with some solvents during the extraction step. Tablets or other solid samples are simply crushed using grinders or knives. Liquid samples, such as beverages, wines, and biological samples are usually first thoroughly mixed, filtered, and/or centrifuged, before further preparation steps, often involving hydrolysis with HCl at room temperature or above. 

Currently, many different techniques are used to isolate GA. An overview of the representative GA isolation conditions is presented in Table 3. These include classical approaches, such as distillation [109], maceration [88,89,90,91,92,94,97,100,101,103,106,108,109,112,116,118,120,123], reflux extraction [27,93,103,106,107,114], reactive extraction [102], enzyme-assisted or polyamide membranes extraction [101] as well as newer and more advanced ones, such as ultrasound-assisted extraction [98,99,115,122], microwave-assisted extraction [103,111], and simpler and miniaturized techniques such as LLME [118], SPE [114], or MSPD [113].

The above brief overview shows that extraction is the most commonly used method. Broadly speaking, there are two main categories of this technique: liquid–liquid extraction (LLE), used to extract liquid from a liquid, and solid–liquid extraction (SLE), the so-called extraction by leaching, used to isolate compounds from solids. Regardless of the category, they can be further divided into conventional extraction techniques, such as LLE and extraction under reflux or maceration, and modern ones, both sophisticated and simplified. The traditional methods are based on the use of the extracting power of various solvents, although in the case of the GA extraction, these are usually water–alcohol mixtures, the application of high temperature, and/or mixing. An analysis of the data presented in Table 3 proves that maceration is the most commonly applied classical extraction technique. Generally, it requires long extraction times and large amounts of organic solvents. It should be added that the maceration time depends on the type of plant matrix and in general, the harder the matrix, the longer the maceration time. For example, Carvahlo et al. [89] found that maceration of GA from *Schinus terebinthiofolius* requires 5 days. In [92], the maceration time of 72 h was used for the complete extraction of GA from the stem bark of *Schinopsis brasiliensis.* Moreover, in the case of *Emblica officinalis* fruit extraction described in [107], a 30-hour maceration was used. Some researchers use the addition of butylhydroxytoluene (BHT) to the hydroalcoholic mixture to stabilize the sample during a longer maceration time [115]. In order to increase the efficiency of maceration, the same portion of plant material is subjected to several extraction cycles, usually 2–3 cycles, each time with a fresh portion of the solvent. Another applied solution is the use of ultrasound [98,99,116,123] and/or a magnetic stirrer [108]. To increase the selectivity of the obtained macerates, scientists sometimes use LLE or backward extraction, where they add a small amount of water/organic solvent to the resulting organic/aqueous extract and repeat the extraction process with a given extractant or purify the extract by means of solid phase extraction (SPE) [96,112,114,115,117].

Extraction under reflux is the second, after maceration, classical technique of obtaining GA from plant matrices. The use of a higher temperature under the reflux extraction undoubtedly reduces the extraction time [27,93]. Nevertheless, it should be mentioned that application of a higher temperature for a few hours can lead to an analyte loss, it increases energy consumption and costs, and has negative environmental impacts from the chemicals’ disposal. Additionally, even a trace amount of organic solvent can be a problem if present in the final product, especially in food and pharmaceutical applications [53].

#### 5.1.2. Modern Extraction Techniques

The simplicity of traditional techniques makes them still commonly applied, despite their above mentioned disadvantages. Yet, they make it necessary to consider the use of new, more ecological, and environmentally friendly GA extraction techniques [126]. These innovative extraction techniques of gallic acid, which not only eliminate the errors of classical approaches but also improve the isolation process, include, among others, ultrasound-assisted extraction, microwave-assisted extraction, or the MSPD procedure [98,99,103,113,116,123]. By reducing the use of mostly toxic organic solvents, these techniques could improve the quality of the extracts. In addition, the MSPD technique allows for the proper study of plant composition [127,128,129,130,131,132]. This is due to the fact that it does not induce any transformation and/or degradation processes in the analyzed substances, and consequently allows one to determine the actual concentration of phenolic compounds to which gallic acid belongs. As a consequence, modern extraction techniques can be implemented on both the industrial and laboratory scale [126].

Ultrasound-assisted solvent extraction (UASE) offers an alternative to conventional extraction techniques. The process uses the cavitation phenomenon, generating macroturbulences, high-speed intermolecular collisions, and disturbances in the microporous particles of natural materials, which accelerates diffusion and improves the mass transfer. Due to the possibility of using this phenomenon to shorten the extraction time and increase the yield of thermosensitive compounds at lower processing temperatures, there is an increasing interest in the use of ultrasound for plant extraction. UASE provides the greater opportunity of increasing extraction ability. In addition to the careful selection of an appropriate solvent inherent in the conventional techniques, the process can be further optimized, which is an important part of the UASE process [98]. The optimization takes into account ultrasound frequency, amplitude, the number of extraction cycles, exposure time, and nominal output power. Another advantage of UASE, which is equally important, is the low cost of the equipment necessary for this technique because the process is most often carried out in the ultrasonic baths found in every laboratory. The ease of use and security add to the appeal of this approach. For example in [99], the UASE process was used to extract GA from the fermented Triphala waste of *Aspergillus niger* using deionized water as an extraction medium at 30 °C, showing that, at 40 kHz ultrasonic frequency, the GA extraction yield, compared with the yields from water extraction without ultrasonic assistance, increased from 0, 25 ± 0.03 to 1.26 ± 0.25 mg/g with a shorter extraction time, from 60 to 30 min. In turn, in [133], examining the best UASE parameters of gallic acid from *Chromolaena* sp., it was shown that a sonication time of 80 min with 90% power gives the maximum yield of the compound. Another research group [98] optimizing the parameters of the UASE process, i.e., the effect of extraction time (5–60 min), temperature (30–75 °C), sonication power at 37 kHz (30–90%), pH (2–10), and the ratio of solids to solvents (1:5–1:40 g/mL) for the GA extraction from *Ficus auriculata* leaves, obtained the maximum yield after 30 min of the process at 50% power, using the solids to solvent ratio 1:10 g/mL at 50 °C. In the cited paper, of the various tested solvents, 50% methanol gave the highest extraction, followed by the alkaline water (pH 8) and 50% ethanol, where the gallic acid content of the extract was found to be 329.46 mg/L, 284.16 mg/L, and 183.74 mg/L, respectively.

In another approach, the SLE process of GA was enhanced by microwave heating based on the direct absorption of microwave energy by dipoles and ions and its conversion into thermal energy as the microwaves pass through the medium. In other words, the polarity of molecules within the solid samples causes resistance to movement or friction with each other, resulting in heat that affects the plant cells and causes the extraction of substances. In MASE, the effectiveness of the extraction can be affected by the frequency and power of microwaves, duration of irradiation, moisture content, and particle size of plant samples, type and concentration of solvent, ratio of solid to liquid, extraction temperature, and number of extraction cycles. Of these factors, the solvent is regarded as one of the most important parameters, which affects not only the solubility of compounds but also the absorption of microwave energy. There are few papers devoted to the application of the MASE technique for the extraction of GA from solids. One of them found that GA was extracted rapidly from *Acacia arabica* bark using 20% MeOH [103]. In turn, in [111], MASE was successfully applied to extract GA from the leaves of a Eucalyptus hybrid.

The above literature review shows that most of the extraction methods use organic solvents, which affects the high cost of the extracted product and has a negative impact on health and the environment. However, recently there have been some reports on the possibility of using ionic liquids (ILs) in tandem with microwave energy as an effective agent for GA extraction. Composed of large and unsymmetrical organic cations and organic or inorganic anions, ILs are liquid molten salts at temperatures below 100 °C. In addition to the solvation capacity for a wide range of compounds, the most aprotic ILs are distinguished with chemical, thermal, and electrochemical stability, non-flammability, and negligible volatility, which reduces both the environmental impact and the process cost. They have also been recognized as tunable solvents, which results from the large number of ion combinations and the possibility of designing fluids with a specific task. In [134], for the isolation of GA from *Eucalyptus globulus* leaves, the Brönsted acidic ionic liquid-based microwave-assisted simultaneous hydrolysis and extraction method (BMSHE) was used. In the approach, [HO_3_S(CH_2_)_4_mim]HSO_4_ was used as the hydrolysis catalyst as well as the extraction solvent. The optimized parameters were as follows: [HO_3_S(CH_2_)_4_mim]HSO_4_ concentration of 1.0 M, liquid–solid ratio of 30 mL/g, microwave irradiation time of 20 min, and microwave irradiation power of 385 W. According to the authors of the cited paper, the proposed method is a greatly efficient, time-saving, low-energy, and eco-mild methodology that may become a new potential candidate for obtaining bioactive compounds from plant materials.

Concluding the review of modern gallic acid extraction techniques, it is worth mentioning the MSPD procedure, which is one of the most promising solid sample extraction techniques. Briefly, this method involves the dispersion of the sample over a solid support followed by elution of the released compounds with a relatively small volume of solvent. It offers several advantages over other sample preparation methods, including very low cost, simplicity, and exceptional isolation performance, comparable to much more sophisticated methods [135]. In [113], the MSPD method was developed to extract GA simultaneously with 22 other phenolic compounds from wine samples. The optimized MSPD procedure required a small volume of wine (1 mL), commercially available silica gel (1.5 g) as the solid-phase dispersant, and a small volume of ethyl acetate (5 mL) as the eluting solvent. Under these conditions, after a short dispersion time (15 min), the mean values of recoveries ranged from 87% to 109% with great repeatability (RSD < 9%), and detection limits <8 μg/L confirmed the usefulness of the proposed methodology.

### 5.2. Chromatographic and Non-Chromatographic Methods of GA Determination

As mentioned, chromatography is currently the preferred method for direct determination of gallic acid in various types of samples. The examples of currently applied methods of GA analysis are summarized in Table 3. The most widely used analytical separation technique is liquid chromatography (LC), in both the form of column chromatography and planar chromatography. In the first case, the stationary phase is in the form of packed column, in which the entire volume of the tube is filled with a sorbent. In the latter, unlike column chromatography, the stationary phase is placed on a plane, often on an aluminum sheet support. The information on the use of gas chromatography (GC) for the GA analysis can also be found in the literature. However, GC requires both high volatility and thermal stability of the compounds to be assayed. The fulfilment of these requirements is associated with the need to change the properties of the tested compounds in the derivatization procedure applied during the stage of sample preparation for the analysis, which of course additionally complicates and extends the analysis time.

#### 5.2.1. LC Separations

Determination of gallic acid by column chromatography is most often performed using high-performance liquid chromatography (HPLC) [26,29,64,83,88,89,90,91,92,93,94,95,96,97,98,99,100,101,102,103], although its more advanced form, i.e., ultra-performance liquid chromatography (UPLC), is also used [28]. These techniques allow the determination of very low analyte concentrations in the presence of sometimes many co-eluting components, often with the properties very similar to these of GA. Their great advantage in the analysis of compounds in plant and biological matrices is the wide range of commercially available columns, which allows one to control the selectivity of the separation process depending on the needs of a given analytical challenge and, owing to the high separation efficiency, the use of smaller amounts of solvents. It should also be remembered that liquid column chromatography does not usually require derivatization of the substances to be determined because, due to the natural ability of most biologically active compounds of natural origin to absorb electromagnetic radiation, separations are most often monitored using a UV [90,91,92,93,94,95,96,97,98,99,100,101,103] or DAD [88,99,102] detector. Considering all the above unique properties, HPLC is recognized as one of the standard methods for the analysis of many compounds, including gallic acid analysis.

The analysis of the data in Table 3 leads to the conclusion that the HPLC analyses of gallic acid are most often performed in the reversed phase system. The most commonly used stationary phases are based on the octadecylsilane phase (C18), typically packed into the 250 mm columns to ensure adequate separation efficiency of natural multi-component mixtures [88,89,90,92,93,94,95,96,98,99,102]. Shorter columns such as 150 and 100 mm packed with finer 2-micron solid phase particles are used in UPLC [28]. Their advantage is better resolution and greater sensitivity achieved in a shorter analysis time. The mobile phase consists of methanol-water or acetonitrile-water mixtures. To increase the retention of GA and suppress its ionization, small amounts of acetic, trifluoroacetic, or formic acid are added to the mobile phase in an attempt to maintain the pH of the mobile phase just below the pKa of the gallic acid [88,89,90,94,96,99,102]. In those separations where the mobile phase component B is methanol, phosphoric acid is also used as a mobile phase modifier [92,93,100,103]. The addition of formic acid, apart from reducing the tailing effect of the analyte peak in the chromatogram, also allows one to increase the ionization efficiency in the source of the mass spectrometer used as a detector [28]. In some applications, to improve the separation of structurally similar compounds, an acidic modifier is added not only to the A component of the phase, but also to the organic component (component B) of the mobile phase [89,90]. Separations are usually performed under gradient elution conditions, although isocratic elution is also used for the analysis of simple matrices [28,91,93,97,98,99,101,102]. As already mentioned, the detectors used to monitor GA separation are most often UV detectors recording at 270 or 280 nm [90,91,92,93,94,96,97,99,102,103] or with a diode array (DAD) [88,99,102], allowing for the collection of absorption spectra in the range from 200 to 400 (600) nm. Some studies use non-selective wavelengths such as 214 nm or 254 nm [96,101,105]. Others, on the contrary, use mass spectrometers to increase selectivity. UHPLC separations are usually monitored with a mass spectrometer to take full advantage of the effective performance of the analytical columns. Ionization, in the context of GA analysis by LC-MS, is performed by electrospray ionization (ESI) [28].

According to the literature study and the data presented in Table 3, the other branch of liquid chromatographic methods applied for GA analysis is planar chromatography, also called thin-layer chromatography (TLC) [104,105,106,107,108,109,110,111]. A more advanced form of the process is called high performance thin-layer chromatography (HPTLC). This technique overcomes the significant TLC hurdle as it enables quantification of compounds. Another advantage of HPTLC is greater resolution and accuracy when compared to TLC. For this reason, it is seen as a more professional, robotic version of TLC where manual handling is reduced, thus also saving time. When it comes to the basics of the process, HPTLC is not much different from TLC. Briefly, the sample dissolved in a volatile solvent is applied to a sorbent deposited on a flat and inert plane (usually glass) and separated based on the differences in polarity of the sample compounds by immersing the plate in the specific solvent system. The mobile phase migrates up through the plate and provides a carrier for the components of the analyzed sample. The solvent moves up the plate together with the sample via the capillary action. In the mixture, components move up the plate at various rates due to the differences in their partitioning behavior between the mobile liquid phase and the stationary phase. Various derivatization reagents are used to visualize spots of colorless compounds, but in the case of acids these are most often pH indicators, such as bromophenol blue. The difference is that HPTLC uses a computer system connected to an automated sampler and scanner. An automatic sampler applies a predetermined amount of sample on the plate, after which the plate can be developed in a glass chamber or automatic developing chamber. Finally, the plates are immersed in the derivatization reagent either manually or using a dipping device. Alternatively, the derivatizing agent can be sprayed on the plate using a derivatizer. Finally, the compounds are quantified by scanning the plate with a scanner. In the case of GA analysis by HPTLC, densitometric visualization of the chromatographic spot at 254 nm and 366 nm using deuterium and mercury lamps is used more often [104,105,106,108]. HPTLC analyses are conducted in the normal phase system unlike the typical analyses made by reversed phase column liquid chromatography. The stationary phase is the silica gel, whose surfaces are rinsed with methanol before starting the analyses, and then activated by heating at 60 or 120 °C for a few or several minutes. A typical mobile phase is a mixture of toluene, ethyl acetate, and formic acid mixed in various volume ratios [104,105,106,107,108,109,110,111].

The analytical capabilities of the liquid chromatographic systems are a specific combination of the resolution capabilities of the stationary and mobile phases and the sensitivity and selectivity of the currently applied detectors. The quantification of GA in the complicated and complex samples requires column chromatography and possibly the most sensitive detection methods. However, when the purpose of the analysis is to confirm the presence of gallic acid in a well-known type of material, the HPTLC approach is usually chosen. For this reason, this approach is now proposed as a method to standardize the quality of herbs and related products. In both of these methods, the assessment of GA content in the tested samples is usually performed by the absolute (external) quantification method (the so-called external calibration method), using a calibration curve prepared from the standard solutions of test substances with several concentration levels. An alternative is the method of relative (internal) quantification (the so-called internal calibration method), characteristic of column chromatography. In this approach, the calibration curve obtained for the solutions of the tested substances, but this time enriched with the addition of another substance, the so-called internal standard with properties similar to those of the analyte, is used. The addition of a known amount of the internal standard to the sample before its preparation stage also compensates for the loss of the analyzed substance at particular stages of preparation, making the method more accurate. Moreover, unlike the external calibration method, which is based on the signal intensity of the analyte itself, the internal calibration method uses the ratio of the signals, i.e., the signal of the analyte to the signal of the internal standard, making this method also more precise. This explains why column liquid chromatography is the technique of choice for accurate and precise GA analysis in complex natural sample types.

#### 5.2.2. GC Separations

As already mentioned, the use of the GC technique for the analysis of gallic acid involves the need to derivatize the analyte. This is to enable the analysis by increasing the volatility and thermal stability of GA, but also to improve the peak shape by regulating interactions with the chromatography column and reducing surface adsorption. By analyzing the data collected in Table 3, it can be seen that, in the case of gallic acid, derivatization is performed based on the silylation reaction [112,113,114,115,116,117,118].

Silylation is the most commonly used derivatization method in GC. The reaction mechanism is based on the nucleophilic attack on the silicon atom in the silylating reagent and consists of introducing a silyl group into the analyte molecule in place of the active hydrogen. Replacing the active hydrogen with a silyl group reduces the polarity of the compound and hydrogen binding. The choice of a silyl reagent is based on its reactivity and selectivity for the compound, the intended use, the stability of the derivative, and the abundance and nature of the reaction by-products. Nevertheless, as can be seen in Table 3, N,O-bis(trimethylsilyl)trifluoroacetamide (BSTFA) is a commonly used reagent to introduce the trimethylsilyl (TMS) group into the GA molecule. It is used alone or in the presence of a catalyst such as trimethylchlorosilane (TMCS) [111,114,115,116,117] or pyridine [113,118]. TMCS is often added to the reagents to increase the potency of the silyl donor. In turn, pyridine is used as a non-protic solvent and catalyst because it can act as an HCl scavenger in the organosilane reactions.

Silyl derivatives are usually sensitive to moisture, which degrades both TMS reagents and derivatives. To prevent this, in the sample preparation stage, the use of water as an extractant or component of the extraction mixture is eliminated in favor of readily removable low-boiling organic solvents such as ethyl acetate [113,114,118]. In the other approaches, the extract is purified on the diatomaceous earth by SPE or repeated evaporation of the solvent to dryness, possibly using azeotropic removal of water from the extract by adding DCM [115]. Using the excess derivatization reagent and solvent can also help minimize problems with moisture or other sample contaminants. In some studies, in order to accelerate the reaction, heating was used at a temperature of 35 to 100 °C for a period of several minutes to 8 h [114,115,116]. As can be seen in Table 3, the GC separations are carried out on the DB-1 column or the slightly more polar DB-5 column or its equivalents with a programmed change in the column temperature. The sample is most often introduced in the splitless system [114,115,116,117,118], the split system is used less often, and the split ratios found in the literature in the latter case are 1:10 [112] and 1:50 [113]. Bearing in mind the negative impact of silyl derivatives on the flame ionization detectors, mass spectrometers are used as detectors where the addition of a silyl group gives either more interesting diagnostic fragments or characteristic ions used for SIM (Selected Ion Monitoring) [14,114,118].

#### 5.2.3. Non-Chromatographic Methods of GA Determination

In order to present the available and currently used analytical tools for assessing the content of GA in various types of samples, attention should be paid to the electrochemical methods.

Recently, there has been an increase in the interest of researchers in the possibilities of various electrochemical methods, measured by the number of published papers on the determination of gallic acid. These include voltammetric, amperometric, and polarographic techniques (see Table 3). Electrochemical techniques pay more attention to the electrochemical oxidation of hydroxyl groups in gallic acid [119]. The advantage of these methods over the other methods that are characterized by expensive equipment, complicated operation, time consumption, sample preparation and a large amount of toxic organic solvents is huge [120,122]. Electrochemical methods are considered to be more selective, sensitive, cheaper, and with a shorter response time, as well as being easy and simple. Moreover, the problem of low kinetics and high potential necessary for the oxidation of gallic acid can be disregarded owing to the rapid development of nanoscience. The use of nanomaterials for the construction of electrochemical sensors is of great importance. Owing to their specific chemical and physical properties, electrochemical methods become more convenient in the determination of the tested compounds. The modified electrodes are a powerful tool for environmental, clinical, and food analyses as well as gallic acid determination. Table 3 presents the examples of electrochemical methods for the determination of gallic acid in various matrices.

In this review of non-chromatographic methods for the analysis of gallic acid, rectitude also requires at least a mention of chemiluminescent techniques that exploit the luminescent properties of compounds (for example: cadmium sulphide (CdS/T) quantum dots coated with luminol or thioglycolic acid) [136,137], high-throughput capillary electrophoresis and spectroscopy [138]. However, a review of the literature reveals that, currently, these techniques are less frequently used for the determination of gallic acid in natural samples.

## 6. Conclusions

The objective of this review paper is to update knowledge about the occurrence, properties, and methods that have been developed for the extraction and analysis of gallic acid in various materials.

Currently, as shown in the paper, there are many known positive effects of this compound including anti-inflammatory, anti-dengue, anti-platelet, anti-apoptotic, anti-cancer, anti-microbial, anti-oxidant, pro-oxidant, and other effects. Knowledge of these activities and the commercial use of gallic acid, its production or side effects, as well as the various analytical methods used to identify and quantify this active plant ingredient is very important. Recently, more efficient methods of extracting this compound and more efficient and reliable methods of its analysis have been sought. Therefore, more economical and ecological extraction approaches are increasingly based on the assisted extraction techniques in combination with chromatographic methods of analysis, mainly HPLC, especially when gallic acid is present at low concentrations in complex mixtures. This is due to the fact that gallic acid can be a very promising agent in medicine (both as an effective drug and an aid to therapy). The presented knowledge could be helpful in obtaining preparations rich in gallic acid for use in functional foods and nutraceuticals.

## Figures and Tables

**Table 1:** The physical and chemical properties of gallic acid [17,18,19,20].

Property Name	Property Characteristics
Chemical structure	C_7_H_6_O_5_
Molecular weight	170.12 g/mol
CAS no.	149-91-7
Physical State	Solid
Appearance	Fine crystals, white yellowish-white or pale, fawn-color
Melting point	250–253 °C
Odor	Odorless
Solubility in water	12 g/L (20 °C)
Density	1.694 g/cm^3^ (anhydrous)
log P	0.7
PKa	1 (4.09–4.41); 2 (7.30–8.70); 3 (11.45–12.17)
Lethal dose, 50%	5000 mg/kg (rabbit); 4300 mg/kg (rat) (intraperitoneal); 320 mg/kg (mouse) (intravenous)

**Table 2:** The occurrence of gallic acid.

Food and Beverages	Content	References
Raspberry	19–102 mg/kg	[22]
Strawberry	21–89 mg/kg	[22]
Grape juice, black	79 mg/kg	[23]
Grape juice, green	110 mg/kg	[23]
Blackberry	8–67 mg/kg	[24]
Black currant	30–62 mg/kg	[24]
White currant	3–38 mg/kg	[24]
Evening primrose (*Oenothera biennis*)	15 (36) mg/kg	[25]
Hazelnut (*Corylus avellana*)	1 (5) mg/kg	[25]
Witch hazel bark (*Hamamelis virginiana* L.)	bark 0.59 (% *w*/*w*)	[26]
Guava leaf/twig (*Psidium guajava* L.)	twig 0.12 (% *w*/*w*)	[27]
Golden root (*Rhodiola rosea* L)	leaf 0.21 (% *w*/*w*)	[28]
Golden root (*Rhodiola kirilowii* L.)	12.18 mg/g	[28]
Wild liquorice root (*Astragalus glycyphyllos* L.)	31.6 mg/100 g	[29]
Emperor’s candlesticks root (*Cassia alata* L.)	3.85 mg/100 g	[30]
Green chiretta (*Andrographis paniculata* L).	0.4 mg/g	[30]

**Table 3:** The review of representative conditions for gallic acid isolation and analysis by HPLC, HPLTC, GC, and electrochemical methods in various solid and liquid matrix types.

Material	Sample Preparation	Analytical Conditions	Ref.
Tea samples, including fermented (black and red), semi-fermented (oolong), and non-fermented (green) teas of different geographical origins	Grinding of samples; triple maceration with 20 mL of 80% (*v*/*v*) methanol (MeOH) for 3 h and then twice with 20 mL of 80% (*v*/*v*) MeOH containing 0.15% HCl for 3 h; filtration of the obtained extracts	HPLC-DAD; C18 column (250 × 4.6 mm I.D, 5-μm); mobile phase: water-acetic acid, 97:3 *v*/*v* (A), MeOH (B); gradient elution program: 100% solvent A for 1 min, followed by a linear increase in solvent B to 63% in 27 min; flow rate: 1 mL L/min; detection: λ = 200–400 nm	[88]
*Schinus terebinthifolius* Raddi bark	Maceration in 40% ethanol (EtOH) over 5 days; extracts hydrolysis by refluxing for 1.5 h with 5% sulfuric acid, followed by LLE with 30 mL of ethyl acetate (EtAc) and concentration in a rotavapor	HPLC-DAD; C18 column (250 × 4.6 mm, 5 μm); mobile phase: ACN:H_2_O and MeOH:H_2_O at pH 2.7(adjusted with formic acid (HCOOH)); gradient elution I: organic phase changes from 5 to 80% for 20 min; gradient elution II: organic phase changes from 15 to 80% for 20 min; flow rate: 1 mL/min; detection: λ = 200–400 nm	[89]
Stem bark of *Q. parviflora* and *Q. grandiflora*	Maceration with a hydroalcoholic solution; complete evaporation of the solvents under reduced pressure at 40 °C	HPLC-UV; C18 column (250 × 4.6 mm., 5 μm); mobile phase: water (+0.05% trifluoroacetic acid [TFA]) as solvent A and MeOH (+0.05% TFA) as solvent B; gradient elution profile: 0–12 min (15–40% B); 12–14 min (40–74%) B; 14 -16 min (74–15%) B, 16–18 min (15% B); flow 0.8 mL/min; λ = 280 nm	[90]
Fresh fruit of *Benincasa hispida* (Bh)	Homogenization of pulps; low heating maceration with water at 60 °C for 30 min followed by drying for 2 days at 55–60 °C	HPLC-UV; LiChrospher 100 RP-18 (125 × 4 mm, 5-μm); mobile phase: 0.01 M potassium dihydrogen phosphate-ACN (85:15, *v*/*v*) at pH 3.2; flow 0.75 mL L/min.; detection: λ = 280 nm	[91]
Stem bark of *Schinopsis brasiliensis* Engl., Anacardiaceae	Hydroalcoholic maceration of dry powder material with water:EtOH mixture (30:70, *v*/*v*) for 72 h; drying of the obtained extract at 140 °C	HPLC-UV; Phenomenex Gemini NX C18 column (5-μm, 250 × 4.6 mm); mobile phase: 0.05% orthophosphoric acid (A): MeOH (B); gradient program: 90–10% B (10 min), 70–30% B (3 min), 40–60% B (5 min), 60–40% B (3 min), 80–20% B (3 min) and 90–10% B (6 min); flow 1 mL L/min; λ = 271 nm	[92]
*Rhodiola kirilowii* (Regel.) Maximroot *Rhodiola rosea L*. root	Extraction under reflux for 45 min with 70% MeOH with acetylsalicylic acid; evaporation of organic phase and dissolution in the mobile phase	UPLC-ESI MS/MS; C18 column (1.7 μm, 2.1 × 50 mm); mobile phase: MeOH (A), water (B); flow 0.35 mL L/min; isocratic elution: 95% of phase A; column temperature: 24 °C; ion source temperature: 100 °C; desolvation temperature: 300 °C; gas flow rate: desolvation gas: 700 L/h; cone gas: 10 L/h.	[28]
*Herba Gei*	Extraction under reflux with 30% EtOH in a water bath for 90 min; filtration of the obtained extract	HPLC-UV; C18 column (250 × 4.6 mm, 5 μm); column temperature: 30 °C; mobile phase: MeOH (B) -0.1% aqueous phosphoric acid (A); isocratic elution: (10% B, 90% A); flow rate 1 mL/min; λ = 273 nm	[93]
*Syzygium polyanthum* leaf	Maceration with water and MeOH; filtration; evaporation and dissolution of 10 mg of the aqueous and methanolic extracts in 1 mL of MeOH	HPLC-UV; C18 column (250 × 4.6 mm, 5 μm); mobile phase: 0.1% aqueous HCOOH (A) and ACN (B); gradient elution profile: 0–12 min with 15–25% B, 12–22 min with 25% B, 22–25 min with 25–15% B, and 25–30 min with 15% B; flow 1 mL/min; λ = 280 nm	[94]
Fruits mixture (Triphala): Amla (*Phyllanthus emblica* Linn.), Baheda (*Terminalia belerica* Roxb., Fam. Combretaceae), and Harde (*Terminalia chebula* Retz., Fam. Combretaceae)	Triple maceration with MeOH; combination of the obtained extracts; concentration at reduced temperature (50 °C) on rotavapor; filtration through a nylon filter	HPLC-UV; C18 column (250 × 4.6 mm, 5 μm); mobile phase: ACN (A) and o-phosphoric acid in water (0.3%) (B); gradient elution profile: 0–5 min with 90–88% B, 5–6 min with 88–86% B, 6–9.5 min with 86–80% B, 9.5–10.5 min with 80–79% B, 10.5–12 min with 79–78% B, 12–22 min with 78–76% B, and 22–30 min with 76–90% B; flow 0.8 mL/min; λ = 254 nm	[95]
European red oak (*Quercus robur*), North American white oak (*Quercus alba*), blocks	Grinding of the sample into fine dust; collecting of oak wood dust on the polycarbonate membrane filters; filter maceration with the MeOH/water mixture (80/20 *v*/*v*) for 60 min; evaporation of MeOH; LLE with EtAc–EtOH (95/5 *v*/*v*) and then evaporation and reconstitution in 0.07% HCOOH (100 μL, pH 2.7); filtration through a nylon filter	HPLC-UV; C18 column (150 × 4.6 mm, 5 μm); mobile phase: pH 2.7 HCOOH (A) and 0.07% HCOOH in MeOH (B); gradient elution: 100% (A) for 20 min, 100% to 20% in 5 min, 20% for 10 min, 20% to 100% in 5 min, and 100% for 20 min; flow 0.7 mL/min; λ = 270 nm	[96]
Human plasma and urine	Acid hydrolysis; maceration with EtAc; evaporation of organic phase; dissolution in the mobile phase	HPLC-UV; Chrospher 100 RP-18 column (5 μm; 120 × 4 mm); method 1: mobile phase (4.4 × 10^−3^ M phosphoric acid in water); flow 1 mL L/min; λ = 220 nm and 270 nm; method 2: mobile phase water–ACN (97.5:2.5, *v*/*v*) modified with 0.025% phosphoric acid; flow 1 mL L/min; λ = 220 nm and 270 nm	[97]
*Ficus auriculata* Lour. leaves (Roxburgh Fig)	UASE with the aqueous solvent at 37 kHz; centrifugation of the extract at 10000 rpm for 10 min; filtration using Whatman filter paper	HPLC-IR-UV-Vis; Shimpack C18 column (250 × 4.6 mm, 5 μm); mobile phase consisted of pure ACN (A) and 0.1% ortho-phosphoric acid in water (B) with a stable composition of 20% A and 80% B; flow 0.8 mL L/min	[98]
Triphala waste	Grinding of the dried sample into small particles; fermentation with *Aspergillus niger;* drying and milling of the fermented sample into powder; UASE with water (10–60 min, 40 kHz at 30 °C)	HPLC-UV; C18 column (250 × 4.6 mm, 5 μm); mobile phase: ACN (A) and acetic acid in water (0.1%) (B); gradient elution; flow 1 mL/min; λ = 280 nm	[99]
Fruit wines and grape wines of Papazkarasi-type cultivar	Removing the alcohol using a rotatory evaporator; lyophilization of residues; dissolution in water	HPLC-UV; C18 column (150 × 4.6 mm, 3.5 μm); mobile phase: 10 mM phosphoric acid as solvent A and MeOH as solvent B; gradient elution profile: 0–15 min (0–60% B), 15–20 min (60–80% B), 20–22 min (80–100% B), 22–27 min (100–0% B), and 27–32 min (0% B); flow 1 mL/min; λ = 214 nm	[100]
*Camellia sinensis* leaves	Maceration with hot water and then polyamide membrane separation; filtration with filter paper	HPLC-UV; C-18 column; mobile phase, consisting of 7% (*v*/*v*) solvent A (100% ACN) and 93% of solvent B (20 mM KH_2_PO_4_); flow 1 mL/min.	[101]
Aqueous solutions	Reactive extraction with tri-n-caprylylamine in hexanol (0.234 mol/L) at 25 °C; filtration through a syringe PVD filter	HPLC-DAD; Eclipse XDB-C18 column (250 × 4.6 mm, 5 µm); at 35 °C; mobile phase: ACN (10%) and 0.2 mole of the aqueous solution of acetic acid (90%); flow 1 mL/min; λ = 273 nm	[102]
*Acacia arabica* bark	Grinding; MASE and reflux extraction with 20% MeOH at T = 88 °C for 1–7 h; maceration in 20% MeOH for 12–30 h; centrifugation of the obtained extract	HPLC-UV; C18 column (150 × 4.6 mm, 5 μm); mobile phase: 0.025% o-phosphoric acid in water (A) and MeOH (B); gradient elution profile: 0–5 min, 20% B; 5.1–15 min, increasing gradually from 50% to 80% B; 15.1–18 min, 20% B; flow 1 mL/min; λ = 272 nm	[103]
Mixture of Vidanga (*Embelia ribes* Burm.), Amalaki (*Emblica officinalis* Geartn), Haritaki (*Terminalia chebula* Retz.), Nishotha or Trivrt- Root (*Operculina turpethum* Linn.), and Guda (*Jeggary*)	Maceration with MeOH; evaporation; reconstitution in MeOH	HPTLC with silica gel 60 F254 plates (10 × 10 cm, 0.2 mm thickness); mobile phase: toluene/EtAc/MeOH/HCOOH (5: 4: 0.5: 0.5, *v*/*v*); migration distance: 80 mm; λ = 254 nm	[104]
Amrtottara kvatha mixture containing fresh stem of *Tinospora cordifolia* (Willd.) Miers (Guduchi), dried fruit rind of *Terminalia chebula* Retz (Haritaki), and dried rhizome of *Zingiber offficinale* Roscoe (Shunti)	Maceration with boiling water; evaporation at 80 °C for 2 h; reconstitution of the residue in MeOH	HPTLC with silica gel 60 F254 plates (20 × 20 cm) with the aluminum sheet support; the mobile phase: toluene/EtAc/HCOOH (5/7/1, *v*/*v*/*v*); migration distance: 70 mm; λ = 254 nm and λ = 366 nm	[105]
Polyherbal tablets (Amalant and Sookshma Triphala Tablet) containing *Embelica Officinalis*	Extraction under reflux with 50 mL of MeOH; filtration of the obtained extract	HPTLC with silica gel 60 F254 plates (20 × 10 cm with 0.2 mm thickness); mobile phase: toluene/EtAc/HCOOH (6/3/1, *v*/*v*/*v*); distance: 60 mm; λ = 254 nm	[106]
Flower buds of *Syzygium aromaticum* (L.) Merr. & Perry (clove)	Extraction under reflux with MeOH; concentration of the obtained extract to a known volume	HPTLC with the silica gel 60 F254 plates (20 × 10 cm, 0.2 mm thickness); the mobile phase: toluene/EtAc/HCOOH (10.8 mL) (3:2:0.4, *v*/*v*); distance: 80 mm; λ = 280 nm	[107]
Selected polyherbal supplements	Maceration in MeOH enhanced by shaking for 4 hrs using a magnetic stirrer; filtration of the obtained extract	HPTLC with the aluminum backed TLC plate coated with the 0.2 mm layer of silica gel (10 × 10cm); mobile phase: toluene/EtAc/HCOOH (5:5:1, *v*/*v*/*v*); λ = 254 nm	[108]
*Emblica officinalis* fruit	Double maceration with MeOH for 24 h at 25 °C; filtration and concentration of the obtained extract	HPTLC with silica gel 60 F254 (4 × 10 cm); mobile phase: toluene/EtAc/HCOOH at the ratio (7/5/1, *v*/*v*/*v*); λ = 273 nm	[109]
Polyherbal formulation, psoriasis tablets with *Azadirachta indica* Linn., *Curcuma longa* Linn., *Rubia cordifolia* Linn., *Tinospora cordifolia* Willd., *Acacia catechu* Wild and others herbs	Distillation with MeOH; filtration; concentration of the obtained extract to a dry residue; reconstitution in MeOH	HPTLC with silica gel 60 F254 TLC plate (0.2 mm thickness); mobile phase: toluene/EtAc/formic acid (4.5: 3:0.2, *v*/*v*/*v*); λ = 366 nm	[110]
Eucalyptus leaves	Grinding of dried leaves; MASE with different solvents: n-hexane, DCM, EtAc, acetone, MeOH, MeOH/water (60:40, *v*/*v*); concentration and dissolution of the dry residue in MeOH.	HPTLC with silica gel 60 F254 plates (20 × 20 cm) with aluminum sheet support; mobile phase: EtAc/CHCl_3_/formic acid (50:50:3, *v*/*v*/*v*); migration distance: 30 mm; λ = 288 nm	[111]
*Ceratonia siliqua* wood	Maceration with MeOH/water mixture; evaporation; LLE of the aqueous phase with petroleum ether (2 × 25 mL), then with Et2O (2 × 25 mL), and finally with a mixture of Et2O:MeOH (9:1; 2 × 25 mL); hydrolysis with HCl (6 M) in MeOH (1:1, *v*/*v*) at 100 °C for 8 h; evaporation; reconstitution in water and evaporation again (four or five times); LLE with a mixture of Et2O:MeOH (9:1; 2 × 25 mL) and water (2 × 25 mL); derivatization with trimethylchlorosilane (TMCS) and bis-(trimethylsilyl)-trifluoracetamide (BSTFA) (1:3)	GC/MS; DB-1 fused silica capillary column (30 m × 0.25 mm I.D., 0.25 µm film thickness); carrier gas: helium; flow 1.5 mL/min; temperature of the injector: 280 °C; volume of sample: 1µL; split ratio: 1:10; temperature of the interface: 300 °C; column temperature program: oven equilibration time 1 min; initial temperature 90 °C for 2 min, then raised to 290 °C at a rate of 20°C/min and then 5 min at 290 °C, and then to 310 °C at a rate of 4 °C/min and kept for 10 min; ionization energy: 70 eV	[112]
Red wine samples	MSPD of wine samples (1.5 mL) acidified to pH 1.0 with 0.1 mL of 1 M solution of HCl and salted with 0.4 g of NaCl using 1.5 g of silica gel (70–230 mesh) as a dispersant and 5 mL of EtAc as the eluting solvent; evaporation of the extract; derivatization with 100 µL of BSTFA and pyridine (1 mL); the procedure gave mean recoveries between 87 and 109% with RSD < 9%	GC/MS in the selective ion monitoring (SIM) mode; the DB-5MS fused silica capillary column (30 m × 0.25 mm, 0.25 µm film thickness); carrier gas: helium; flow 2.5 mL/min; column head pressure 26.04 psi; temperature of the injector 320 °C; volume of sample 1 µL; split ratio 1:50; temperature of the interface 280 °C; column temperature program: oven equilibration time 1 min, initial temperature 120 °C for 3 min, then raised to 292 °C at a rate of 5 °C/min and then to 320 °C at a rate of 30 °C/min with a final isotherm of 2 min; ionization energy: 70 eV	[113]
Balsamic vinegar from Modena	Dilution of the sample with water (1:1); SPE 1) with 1 g diatomaceous earth cartridges using 6 mL of EtAc or n-butanol or isopentyl alcohol or 4-methylpentan-2-one; SPE 2) in the polyamidic SPE cartridge conditioned with 2 mL of MeOH and 2 mL of water and eluted with 3 mL of EtAc; evaporation; derivatization of 1 mL of sample reconstituted in 1 mL of DCM to ensure removal of water (azeotropic removal of water) with 300 µL of 1:1 BSTFA/pyridine at 70°C for 30 min.	GC/MS with the SIM mode; RTX-5MS fused silica capillary column (30 m × 0.25 mm, 0.25 µm film thickness); carrier gas: helium; flow 39 mL/min; temperature of the injector 260 °C; volume of sample 1µL; splitless mode; temperature of the interface 280 °C; column temperature program: oven equilibration time 1 min; initial temperature 90 °C for 1 min, then raised to 240 °C at the rate of 20 °C/min and then 240°C for10 min, then to 280 °C at a rate of 20 °C/min with a final isotherm of 1 min; ionization energy: 70 eV	[114]
*Origanum dictamnus* (dictamus), *Eucalyptus globulus* (eucalyptus), *Origanum vulgare* L. (oregano), *Mellisa officinalis* L. (balm mint), and *Sideritis cretica* (mountain tea)	Maceration of the dried sample (0.5 g) with 62.5% aqueous MeOH containing BHT (1 g/L); the addition of HCl (10 mL); sonication of the extraction mixture for 15 min and extraction under reflux at 90°C for 2 h; LLE with EtAc (3 × 10 mL); reduction of the organic phase and removing moisture with anhydrous Na2SO4; derivatization after the evaporation of the solvent with the mixture TMCS (100 µL) and BSTFA (200 µL) at 80 °C for 45 min.	GC/MS; capillary column low-bleed CP-Sil 8 CB-MS (30 m × 0.32 mm, 0.25 µm film thickness); carrier gas: helium; flow 1.9 mL/min; temperature of the injector 280 °C; volume of sample 1 µL; splitless mode; temperature of the interface 290 °C; column temperature program: oven equilibration time 1 min; initial temperature 70 °C then raised to 135 °C at 2 °C/min, kept for 10 min and then raised to 220 °C at 4 °C/min and kept for 10 min and up to 270 °C at a rate of 3.5 °C/min with a final isotherm of 20 min; ionization energy: 70 Ev	[115]
Wines from different regions of Poland	UASE-PMLS of 25 µL sample on 60 mg of MgSO4, used as the sample support, using 1 ml of ACN or DCM or EtAc or MeOH; exposition for 25 min in an ultrasound bath; evaporation of the extract to dryness; derivatization with a mixture of 1% TMCS in BSTFA (30 µL) at 35°C for 30 min	GC-MS; ZB-5 MS capillary column (30 m×0.25 mm, 0.25 µm film thickness); carrier gas: helium; flow 1 mL/min; temperature of the injector 240 °C; sample volume 2 µL; splitless mode; temperature of the interface 300 °C; column temperature program: 70 °C for 1 min, then increased to 280 °C at 10 °C /min and kept for 5 min; ionization energy: 70 eV	[116]
Blue Oak Plant	Hydrolysis of sample (1 g) with 10 mL of 3% HCl (*v*/*v*) at 110 °C for 4 h; maceration of hydrolyzed sample after its cooling with 5% EtOH in EtAc (100 mL) and 50 g of Na2SO4; evaporation of the extract to dryness; derivatization with TMCS after the solvent evaporation; LLE cleanup using back extraction with isooctane (1 mL) and water (1:1)	GC-MS; DB-1 capillary column (15 m × 0.53 mm, 0.1µm film thickness); carrier gas: helium; flow 7 mL/min; temperature of the injector 240 °C; sample volume 2 µL; splitless mode; temperature of the interface 280 °C; column temperature program: initial temperature 60 °C for 0.5 min, then raised to 110 °C at a rate of 5 °C/min, and then raised to 180 °C at 10 °C/min, and finally up to 275 °C at 30 °C/min with a final isotherm of 1 min; ionization energy 70 eV	[117]
Wastewater olive oil	LLME with EtAc (0.5 mL) of 2 mL of acidified samples (pH 3) saturated with NaCl; evaporation to dryness in the nitrogen stream; derivatization of the solid residue with 50 µL of a mixture of BSTFA and pyridine in EtAc as the silylation reagent (4:1:5, *v*/*v*/*v*)	GC/MS with the SIM mode; HP-5MS fused silica capillary column (60 m × 0.25 mm × 0.25µm film thickness); carrier gas: helium; flow 1 mL/min; temperature of the injector 250 °C; sample volume 1 µL; splitless mode; temperature of the interface 280 °C; column temperature program: oven equilibration time 1 min; initial temperature 90 °C for 1 min, then raised to 220 °C at 6 °C/min and then to 290 °C at 10 °C/min and kept for 1.23 min and finally to 310 °C at a rate of 40 °C/min and kept for 7.5 min; ionization energy 70 eV	[118]
Green tea sample	Maceration with water at pH3.0, adjusted with a phosphate buffer saline (PBS)	Differential pulse voltammetry with graphene modified glassy carbon electrode used as a working electrode, the saturated Ag/AgCl electrode and a Pt wire, which was used as a reference and counter electrode, respectively; potential window range from −0.4 to 1. 2 V; a scanning rate of 0.1 V·S^−1^; the stirring time −25 s at pH = 3(PBS)	[119]
Red wine	Unprepared samples	Differential pulse voltammetry win zinc oxide nanoparticles modified-carbon paste electrodeas working electrode, a KCl saturated Ag/AgCl as reference electrode, and a platinum wire as counter electrode; potential window range from 0 to 1. 2 V, a scanning rate of 0.1 V·S^−1^; the stirring time −25 s at pH = 2, adjusted with PBS	[120]
Green tea sample	Maceration with water	Differential pulse voltammetry with poly(glutamic acid): graphene modified electrode; potential window range from −0.8 to 2 V, a scanning rate of 0.1 V·S^−1^ at pH 5, adjusted with acetate buffer	[121]
Apple juice, lemon juice, peach juice, green tea, orange juice	Dilution of different beverage samples (5 mL) with 10 mL of phosphate buffer solution (0.1 mol/L, pH 7.0)	Amperometry with silver nanoparticle/delphinid in modified glassy carbon electrode. The experiments were carried out at a potential of 220 mV in a 0.1 mol/L phosphate buffer solution at pH 7.0	[122]
*C. wightii* (*Commiphora mukul*) known as Indianbdellium-tree, *V. agnus-castus* also called *Vitex*, *C. sinensis* (green tea)	UASE with water: EtOH mixture (30:70) at 25 °C at a constant frequency of 35 kHz for 30 min extracts; filtration through Whatman no. 1 filter paper	Differential pulse voltammetry with carbon paste electrode modified with carboxylated multi-walled carbon nanotubes; a potential window range from 0 to 0.9 V, a scanning rate of 0.148 V·s^−1^ and solution was 0.2 M PBS at pH 2.0	[123]
Apple peel, apple flesh, nettle	Maceration and dilution	Differential pulse voltammetry with a three electrode cell including modified or bare CPE, saturated calomel electrode and a platinum wire as working reference and counter electrodes, respectively; a potential window of 0.0–0.4 V; the scan rate was 40 mV s^−1^ and solution was phosphate buffer at pH 7.0	[124]
Fruit juice (e.g., orange, apple, and apricot juice)	Unprepared sample	Differential pulse polarography with dropping mercury electrode as a working electrode, a platinum counter electrode, and an Ag/AgCl (3 M NaCl) reference electrode; optimum conditions: 200–1000 μL of fruit juice samples pH 10.0, at the reduction (peak) potential of –160 mV, 2 s drop time, and 50 mV pulse amplitude	[125]

## Data Availability

Not applicable.
